# Editorial: Host-diet-microbiome interactions in obesity prevention and treatment

**DOI:** 10.3389/fnut.2024.1541977

**Published:** 2025-01-30

**Authors:** Javad Barouei, Mary E. Kable, Mahta Moussavi, Yu-Hsin Hsieh

**Affiliations:** ^1^Department of Agriculture, Nutrition and Human Ecology, Prairie View A&M University, Prairie View, TX, United States; ^2^Cooperative Agricultural Research Center, Prairie View A&M University, Prairie View, TX, United States; ^3^USDA-ARS Western Human Nutrition Research Center, Davis, CA, United States; ^4^Food Security Research Center, Prairie View A&M University, Prairie View, TX, United States; ^5^Division of Medicinal Products, Taiwan Food and Drug Administration, Taipei, Taiwan

**Keywords:** gut microbiome, diet, obesity, omics, probiotics, prebiotics, fermentable carbohydrates, plant bioactive components

Obesity rates have reached epidemic proportions globally, posing significant risks for chronic diseases like cardiovascular disease, type 2 diabetes mellitus (T2DM), and certain types of cancer. The role of the gut microbiome in obesity and its potential as a therapeutic target has gained attention. Understanding the factors that influence the composition of the gut microbiome, especially diet, is crucial for developing strategies to manage obesity. Recent evidence suggests that dietary strategies, such as fermentable carbohydrates, prebiotics, and plant-based bioactive components, benefit metabolic health in obese individuals by modulating the gut microbiome. Consumption of certain microbial cultures (e.g., probiotics) and their products or components has also shown promise in reducing weight gain and improving obesity indicators. However, the precise underlying mechanisms by which these dietary strategies improve the obesity markers remain largely unknown. Recent advances in molecular and high-throughput omic technologies, such as metagenomics, transcriptomics, metabolomics, and proteomics enable a comprehensive investigation of the pathophysiology of obesity and the mechanisms behind potential anti-obesity dietary approaches.

In response to this critical need, this Research Topic collected studies that focused on molecular and omic technologies, along with other advanced tools such as artificial intelligence and machine learning to explore how dietary interventions improve obesity markers via gut microbiome alterations. Here, we overview the findings of five articles published in this Research Topic. Three of these investigated gut-microbiota modulatory and health-promoting effects of plant-based components: stachyose, a prebiotic oligosaccharide (Pi et al.), barley β-glucan (Maruyama et al.), and raspberry leaf polyphenols (Wang et al.). Another study focused on dietary patterns, specifically the Mediterranean diet (Florkowski et al.). Finally, the fifth article explored dietary, and lifestyle digital diet and lifestyle programming based on individuals' microbiome profiles to reduce weight gain (Kumbhare et al.).

The study by Pi et al. investigated the effects of stachyose, a prebiotic oligosaccharide found in legumes, on the microbiota structure and metabolic processes in obese children through simulated *in vitro* fermentation of their fecal samples. Stachyose supplementation increased the abundance of beneficial bacteria like *Bifidobacterium* and *Faecalibacterium*, depleted some harmful bacteria, and improved the production of short-chain fatty acids (SCFAs), particularly acetate, which regulates metabolic health. It also reduced harmful metabolites like hydrogen sulfide and ammonia. These findings suggest stachyose as an early prophylactic dietary intervention to manage obesity in childhood, potentially leading to long-term beneficial impacts on health trajectories. However, as noted by the study investigators, additional research, particularly preclinical and clinical studies, is necessary to reach a more solid conclusion.

Consistent with the beneficial effects of dietary fiber consumption, the study by Maruyama et al. using a machine learning-assisted approach reported a strong association between a complementary symbiotic-like dietary approach and low BMI in Japanese human subjects. This approach involved high consumption of barley, a rich source of the indigestible but fermentable polysaccharide β-glucan, and *Bacillus subtilis*, a glycolytic bacterium found in and essential for the production of natto (a fermented soybean product). The study also observed an increase in the abundance of butyrate-producing bacteria such as *Butyricicoccus* and *Subdoligranulum* in lean individuals. These findings underscore the importance of a diet high in dietary fibers and fermented foods, which may lead to changes in the microbiome composition and function that are favorable for preventing obesity.

Other plant components such as polyphenols, the bioactive compounds with antioxidant and anti-inflammatory properties, have also been shown to reverse obesity-related metabolic dysregulation, possibly by modulating gut microbiota composition and function. In a rodent study, Wang et al. reported that supplementation with polyphenol-rich raspberry leaf extract reduced weight gain, downregulated TNF-α and IL-6 inflammatory signaling pathways, and increased the abundance of beneficial gut bacteria such as *Muribaculaceae, Alistipes*, and *Alloprevotella*. These results suggest that raspberry leaf extract may serve as a dietary supplement with both anti-inflammatory and anti-obesity effects.

Beyond biotic-driven dietary strategies, the Mediterranean diet (MedDiet) has gained attention for its potential to reduce inflammation, a key driver of obesity. The emphasis of the MedDiet on plant-based foods, and healthy fats provides a range of bioactive compounds that support the health of the gut and the whole body. The review article by Florkowski et al. summarized recent studies on the potential anti-inflammatory role of gut microbial metabolites associated with the consumption of a MedDiet. The MedDiet has been shown to positively influence gut microbiota composition, reducing systemic inflammation through the production of SCFAs and other gut-derived metabolites. The high levels of natural antioxidants in the MedDiet have been associated with blunting the proinflammatory effects of bacterial lipopolysaccharides. The MedDiet suppresses pathways associated with inflammation, such as the NOD-like receptor family pyrin domain-containing 3 (NLRP3) inflammasome, and toll-like receptor 4 signaling, which are implicated in the inflammatory responses that drive obesity.

Personalization of dietary interventions is also gaining traction as a strategy to manage obesity by addressing the individual variability in gut microbiota composition. The study by Kumbhare et al. involving a digital health program explored how a tailored diet and lifestyle plan informed by participants' microbiome profiles could influence weight loss. The study identified specific microbial genera such as *Akkermansia, Christensenella*, and *Oscillospiraceae*, and functional pathways including SCFA production, and the degradation of some simple sugars that correlated with BMI reduction. The reported high success of the program reinforces the concept that precision nutrition and lifestyle, guided by microbiome profiles, can be a viable and effective approach to achieving sustainable weight management outcomes.

Collectively, the studies presented employed a variety of designs, including *in vitro*, preclinical, and clinical studies, and also utilized advanced technologies such as omics tools and machine learning approaches to provide insights into the host-diet-microbiome interactions in obesity. These studies highlight the potential of dietary interventions that modulate the gut microbiota to prevent and treat obesity. The potential of diet as a therapeutic tool for obesity management lies not only in individual nutrients but also in understanging the broader network of interactions between diet, microbiome, and host physiology. As we advance our understanding of the role of the gut microbiome in obesity, the integration of microbiome-targeted dietary interventions into personalized nutrition may offer a transformative approach to addressing the obesity epidemic.

